# Assessment of the pathogenicity of *Mycoplasma gallisepticum* and the immunogenicity of its membrane proteins in geese

**DOI:** 10.2478/jvetres-2026-0031

**Published:** 2026-06-30

**Authors:** Xueyan Wang, Baoyi Deng, Ming Li, Weihuo Li, Yangshuo Li, Sheng Yuan, Nan Zhang

**Affiliations:** School of Animal Science and Technology, Foshan University, Foshan 528225, China

**Keywords:** *Mycoplasma gallisepticum*, *Mycoplasma* membrane proteins, *Mycoplasma* pathogenicity

## Abstract

**Introduction:**

*Mycoplasma gallisepticum* (MG) imposes heavy losses on the goose industry in China, yet its goose-specific biology is poorly understood. In elucidation of the biology, the pathogenicity and immunogenicity of MG in geese were analysed.

**Material and Methods:**

Goslings were divided into three groups and either infected with the goose isolate MG-GD01/22, infected with chicken-derived MG R(low) or left untreated as controls. Clinical scores were recorded, and morbidity/mortality, air-sac lesions and tracheal and lung histopathology evaluated. Isolate membrane proteins were separated by SDS-PAGE and screened by Western blot with goose hyper-immune sera.

**Results:**

Both MG-GD01/22 and MG R(low) established infection, with morbidity rates of 90% and 80% and qPCR positivity of 100% and 80%, respectively. The MG-GD01/22 isolate caused 20% mortality and more severe respiratory signs, whereas MG R(low) induced milder disease without deaths. Both strains produced airsacculitis and interstitial pneumonia, but MG-GD01/22 caused more marked pulmonary haemorrhage. Gel electrophoresis showed similar bands at 50–130 kDa with differences at 25–50 kDa. Western blot revealed strong immune responses to conserved 75 kDa and 67 kDa proteins, particularly those of MG-GD01/22.

**Conclusion:**

Both strains are pathogenic in geese, with MG-GD01/22 exhibiting higher virulence. The conserved 75 kDa and 67 kDa proteins represent promising targets for the development of diagnostic assays and vaccines against *Mycoplasma gallisepticum* infection in geese.

## Introduction

*Mycoplasma gallisepticum* (MG) belongs to the Mollicutes class of the *Mycoplasma* genus and is a pathogen capable of causing chronic respiratory disease in poultry. With a size nearly as small as the largest viruses, MG is the smallest self-replicating bacterium (25, 26). Various avian species, including chickens, turkeys, quails, guinea fowls, house sparrows, pigeons, ducks, partridges and geese, are susceptible to MG (5, 14, 19, 28). Infected animals typically exhibit clinical symptoms such as coughing, respiratory rales, conjunctivitis, sinusitis and nasal discharge. *Mycoplasma gallisepticum* can also cause a decrease in egg production, hatchability, weight gain and feed conversion ratio (29). It has been reported that turkeys infected with MG show different symptoms from chickens, which are primarily tearing and infraorbital sinusitis (17). However, the clinical symptoms of MG infection in geese are basically consistent with those in chickens, which establishes that MG mainly damages the respiratory system of geese, causing airsacculitis and exudative tracheitis (27).

Lacking a cell wall structure, MG features soft and pleomorphic cells capable of passing through 0.45 μm microfilters. The outermost layer of MG cells is composed of a capsule, which can be stained with ruthenium red; however, it exhibits low antigenicity. It is not considered a major antigenic target; therefore, research on MG antigens mainly focuses on membrane proteins (12). Numerous studies have confirmed that the VlhA (pMGA), GroEL, PvpA, mgC2, GapA and CrmA MG membrane proteins possess strong immunogenicity and various biological functions (4, 10, 11, 18, 24). Some of these proteins have been successfully used as serological diagnostic antigens and recombinantly expressed antigen vaccines for the diagnosis and prevention of MG in chickens (7, 23, 30).

Previous studies by the present authors have shown that MG is widely prevalent and infects many goose flocks in Guangdong Province, and that MG adapted to geese exhibits significant pathogenicity to them (31). In geese infected with MG, typical symptoms include mouth breathing, head shaking, coughing and rhinitis; in severe cases, the animals may die. *Mycoplasma* infection can also increase the susceptibility of geese to other pathogens, posing a serious threat to the economic sustainability of the local goose industry. Currently, because of the lack of systematic research on the pathogenicity and membrane proteins of goose-associated MG, control measures largely rely on experiences from chicken MG management. Their effectiveness is limited. Moreover, no vaccine is currently available for MG adapted to geese either domestically or internationally, which severely hinders the healthy development of the goose-breeding industry. Therefore, this study aimed to investigate the pathogenicity of goose-derived MG and the immune response elicited by its membrane proteins in geese, and provide a theoretical foundation for effective prevention and control strategies against MG infection in geese.

## Material and Methods

### Strains

The chicken-derived MG reference strain MG R(low) was purchased from the China Institute of Veterinary Drug Control (Beijng, China). The clinical MG isolated from geese by the present authors, MG-GD01/22, is preserved at the China Center for Type Culture Collection (CCTCC, Wuhan, China) with the preservation No. M 20232686.

### Animals

Twelve 45-day-old healthy Wuzong geese (*Anser cygnoides domesticus*) and thirty 7-day-old healthy Wuzong geese were obtained from the goose hatching farm of Qingyuan Red Star Company (Qingyuan, China). All tested gees were raised in the experimental animal room of Foshan University, with free access to food and water. None of the experimental birds showed any respiratory symptoms, and their physical and nutritional conditions remained good. Through careful observation of clinical symptoms and real-time qPCR, it was confirmed that all experimental birds were not infected with MG or carried the MG pathogen, and no signs of infection connected with other diseases were found. All experimental gees were left unvaccinated. The 7-day-old Wuzong geese were randomly divided into three groups, with 10 geese in each group: Group A was infected with goose-derived MG, Group B was infected with MG R(low), and Group C was used as a blank control. Each group was raised in isolation to ensure the accuracy and reliability of the experimental results. Twelve 45-day-old healthy Wuzong geese were randomly divided into two groups of 6 for the preparation of hyperimmune serum. All animal experiments were approved by the Animal Research Committee of the Animal Ethics Committee of Foshan University (Approval No. 205877).

### Establishment of infection model

The MG-GD01/22 strain was reactivated in modified Frey’s medium, passaged to the third generation at a 1:10 ratio, and passaged to the fourth generation for plate counting. The culture was centrifuged at 8,000 rpm at 4°C for 10 min. The supernatant was discarded, and the pellet was resuspended in an appropriate volume of liquid medium (1 mL), ensuring that the bacterial suspension concentration reached at least 109 colony-forming units/mL. The experimental birds were infected intratracheally with a total daily dose of 0.5 mL per bird administered twice a day for three consecutive days. The MG R(low) strain was cultured using the same method and subsequently used for experimental infection in the same regimen.

### Clinical symptoms and morbidity

Clinical signs in the experimentally infected birds were observed daily for 15 d. On the last day, all experimental gees were euthanised humanely, and tissues such as thoracic air sacs, lungs and trachea were collected from all experimental birds under sterile conditions. Extraction of DNA was undertaken from the air sacs for PCR testing to determine the positivity rate.

### Evaluation of airsac lesions

Experimental birds were subjected to necropsy, and pathological changes in the respiratory system were observed and recorded. Air sac lesions were graded pathologically on presence of the specific damage detailed in Table 1.

**Table 1.** Air sac tissue lesion grading after experimental infection of goslings with *Mycoplasma gallisepticum*

Grade	Pathological damage
0	Air sacs appear thin and translucent
1	Air sacs show slight thickening with sparse grey or a few yellow pinpoint exudates
2	Foamy fluid is visible within the air sacs; partial thickening and the presence of grey or yellow exudates are observed
3	Numerous yellow-white caseous exudates are visible, with considerable thickening in most air sacs
4	Nearly the entire air sac is filled with yellow-white caseous exudates and is severely thickened

### Histopathology

The collected tracheal and lung tissues were immersed in 4% paraformaldehyde solution for 3 to 5 d to ensure thorough fixation. After fixation, the tissues were washed under running water for 3 h. Then, the tissues were subjected to a graded dehydration process, then placed in a mixture of ethanol and xylene (1:1) for 30 min and clarified twice in xylene for 30 min each. The tissues were embedded in paraffin using an automatic system, and sections with a thickness of 5 μm were cut with a rotary microtome. The tissue sections were deparaffinised twice in xylene and rehydrated using a series of ethanol dilutions, stained with haematoxylin, and differentiated and rinsed to remove excess stain. Next, the sections were stained with eosin and rapidly dehydrated with graded ethanol. After air-drying, the tissue sections were mounted with neutral resin, labelled and examined under a microscope.

### Extraction of MG membrane proteins

The MG-GD01/22 and MG R(low) strains were cultivated to the third generation at 37°C until reaching the logarithmic growth phase. Cells were then centrifuged at 4°C and 12,000 rpm for 20 min; the supernatant was subsequently discarded. The cell pellet was resuspended in sterile 1× PBS, mixed gently, and washed twice in fresh PBS each wash. Membrane proteins were extracted from both strains using a *Mycoplasma gallisepticum* membrane protein extraction kit (Shanghai Beibo Biotechnology, Shanghai, China), with all steps conducted on ice. The protein concentration was quantified using a bicinchoninic acid (BCA) protein assay kit (Qingdao Lanjieke Biotechnology, Qingdao, China).

### SDS-PAGE

The protein gel was prepared, and electrophoresis was carried out under the initial conditions. The stacked gel was run at 50 V, and the separated gel was run at 70 V. After electrophoresis, the gel was stained using the silver staining kit (Wuhan Seville Biotechnology, Wuhan, China), following the manufacturer’s instructions. The stained gel was then stored in ultrapure water to maintain its transparency. Gel imaging was performed using an automated digital gel imaging analysis system.

### Preparation of hyperimmune serum

Blood samples were collected from each group of six healthy 45-day-old geese to prepare hyperimmune sera of MG-GD01/22 and MG R(low), respectively. This sampling was conducted after clinical observation and qPCR testing confirmed the absence of MG pathogen infection/carriage in the experimental birds. The MG-GD01/22 and MG R(low) strains were passaged to the third generation. Cell cultures were centrifuged at 12,000 rpm for 10 min at 4°C. The supernatant was discarded, and the pellet was resuspended in PBS. This washing process was repeated three times to ensure purity. Extraction of DNA was then undertaken from a portion of the concentrated culture, and its concentration was determined through qPCR and adjusted to 10^10^ copies/μL. The bacterial suspension was then treated with 0.4% β-propiolactone at 4°C for 24 h to inactivate it and verified as free of bacterial growth by plate counting. The inactivated culture and Freund’s adjuvant were mixed in a 1:1 ratio using a syringe to prepare an inoculation. Geese were inoculated by multiple subcutaneous injections on the dorsolateral side of the neck at a dose of 0.5 mL/kg body weight. The primary immunisation was followed by two booster shots with incomplete Freund’s adjuvant, one after three weeks and the other booster shot after another 15 d. Blood samples were collected one week after the last immunisation, and antibody titres were determined using the haemagglutination inhibition (HI) test.

### Western blot

The extracted proteins were separated by SDS-PAGE and transferred onto polyvinylidene membranes. The transfer was performed at 100 A for 70 min. After transfer, the membranes were washed three times in a 10% TBS with Tween (TBST) solution and then immersed in a protein-free blocking solution for incubation. Then, the membrane was incubated overnight at 4°C in a 200:1 dilution of 10% TBST and high-titre hyperimmune serum. The membrane was washed three times in 10% TBST and then incubated with a 4,000: 1 dilution of the secondary antibody working solution in 10% TBST on a horizontal shaker at room temperature. After incubation, the membrane was washed with 10% TBST again. Finally, the membrane was placed in an automatic digital gel imaging analyser, covered with an appropriate amount of working enhanced chemiluminescence solution and imaged by exposure.

## Results

### Clinical symptoms and morbidity

By the third day after infection with the MG-GD01/22 strain, two goslings began to display symptoms including open-mouth breathing, ruffled feathers and reduced food intake. On the fifth day, two goslings from the MG-GD01/22 group died and were immediately necropsied. Subsequently, a majority of the goslings started showing typical open-mouth breathing symptoms around one week later. Four goslings showed severe respiratory distress characterised by frequent neck extension, head shaking and substantial reduction of appetite. On the fifteenth day post-infection, air-sac tissues were harvested from geese during necropsy for DNA extraction and subsequent real-time fluorescence quantitative PCR analysis. The positivity rates and clinical outcomes are detailed in Table 2.

**Table 2.** Clinical outcomes of experimental infection of goslings with a *Mycoplasma gallisepticum* strain previously isolated from geese (Group A, n = 10) or with a strain previously isolated from chickens (Group B, n = 10). Uninfected controls made up Group C (n = 10)

Group	Number of morbidities	Number of deaths	qPCR positivity rate (%)
A	9	2	100
B	8	0	80
C	0	0	0

### Necropsy results

Necropsies were performed on all experimental geese to observe pathological damage within the nasal cavity, trachea, air sacs and lungs. The results indicated that infection with MG-GD01/22 induced significant pathological changes. In the nasal cavity, half of the goslings exhibited substantial yellow, purulent and minor bloody secretions. The goslings displayed minor scattered haemorrhagic spots and viscous exudates in the trachea. Most goslings developed extensive yellow-white caseous exudates in the air sacs, leading to significant thickening and a grey, opaque appearance. There was notable lung tissue congestion, with some goslings also displaying pulmonary emphysema. Examples of these lesions are illustrated in Fig. 1.

**Fig. 1.**
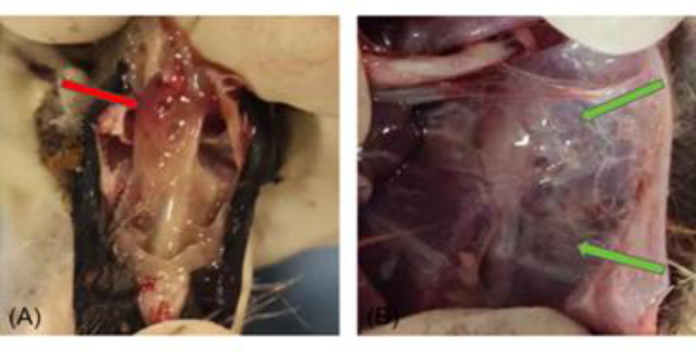
Dissection of gosling experimentally infected with *Mycoplasma gallisepticum*. (A)Numerous inflammatory and minor bloody secretions in the nasal cavity; (B) Yellow-white, cheese-like exudate in the air sacs and markedly thickening and grey discolouration of air sac walls

### Pathological grading of air-sac lesions

The pathological lesion grading results for the air-sac tissue of all experimental geese are recorded in Table 3. Goslings experimentally infected with either the goose-derived MG-GD01/22 or the chicken-derived MG R(low) exhibited significantly higher mean air-sac lesion scores than the uninfected control group. Moreover, goslings infected with the goose-derived strain showed a higher mean lesion score than those infected with the chicken-derived strain, indicating a trend toward greater pathogenicity of the goose-derived isolate.

**Table 3.** Distribution of pathological grades of air-sac tissue after experimental infection of goslings with a *Mycoplasma gallisepticum* strain previously isolated from geese (Group A, n = 10) or with a strain previously isolated from chickens (Group B, n = 10). Uninfected controls made up Group C (n = 10)

Group	Gosling										Mean
	1	2	3	4	5	6	7	8	9	10	
A	1	3	2	3	2	2	1	3	3	3	2.3
B	2	1	3	3	2	2	1	1	2	1	1.9
C	0	0	0	0	0	0	0	0	0	0	0

### Histopathology

Histopathological analysis was conducted on tracheal and lung tissues from both control and infected groups of geese. Tissues were stained with haematoxylin and eosin and examined under a microscope (Figs 2 and 3). Observations in the tracheal mucosa of infected geese included partial loss of cilia from pseudostratified columnar epithelial cells, a decrease in goblet cells within the mucosal epithelium and minor submucosal haemorrhage, with no significant infiltration of inflammatory cells. In the lung tissues, thickening of the walls in several secondary and parabronchi, filled lumens with abundant blood cells and desquamated epithelial debris were noted in *Mycoplasma*-challenged geese. There was also severe vascular congestion and haemorrhage within the pulmonary interstitium, accompanied by lymphocyte infiltration and the formation of lymphoid nodules of varying sizes. Notably, the MG-GD01/22-infected geese group displayed severe vascular haemorrhage in the pulmonary interstitium, which was a pathology distinct from the other infected group. These findings indicate that infections with MG-GD01/22 and MG R(low) in geese lead to interstitial pneumonia characterised by thickened bronchial walls, epithelial cell damage and shedding, luminal haemorrhage and inflammatory exudation in the interstitium, with lymphocyte infiltration forming lymphoid nodules. The pathological impact of MG-GD01/22 was more pronounced than that of MG R(low), especially noting the severe vascular haemorrhage in the pulmonary interstitium of geese, which underscores the higher pathogenicity of MG-GD01/22 in this species.

**Fig. 2.**
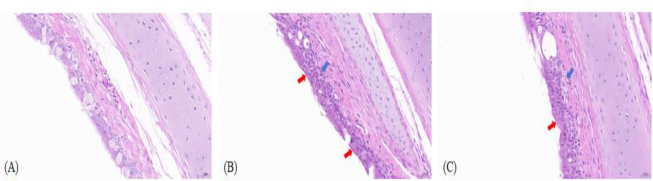
Histopathological lesions in tracheal tissue of uninfected goslings and goslings experimentally infected with *Mycoplasma gallisepticum* (MG). (A) Control goose tracheal tissue (400×); (B) – Goose-derived (MG)-GD01/22-infected goose tissue section (400×); (C) MG R(low)-infected goose tissue section (400×). Ciliated epithelial cell loss (red arrow); submucosal congestion and hemorrhage (blue arrow)

**Fig. 3.**
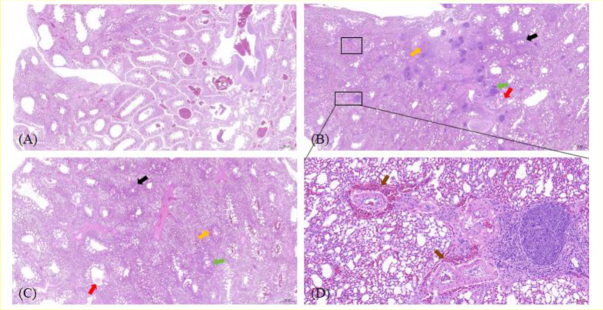
Histopathological lesions in lung tissue of uninfected goslings and goslings experimentally infected with *Mycoplasma gallisepticum* (MG). (A) Control goose lung tissue (20×); (B) MG-GD01/22-infected goose lung tissue (20×); (C) MG R(low)-infected goose lung tissue (20×); (D) MG-GD01/22-infected goose lung tissue (enlarged view of the lower outlined box in (B) 200×). Lymphocyte infiltration foci (green arrow); thickened capillary walls (black arrow); capillary congestion (yellow arrow); pink exudate in secondary and accessory bronchial lumens (red arrow); vascular hemorrhage in the pulmonary interstitium (brown arrow)

### *Mycoplasma gallisepticum* membrane protein concentration results

The absorbance values of MG-GD01/22 were measured at 562 nm. Using the BCA standard curve and the calculation formula, the concentration of MG-GD01/22 was determined to be 1,432.2 μg/mL. The standard curve is shown in Fig. 4.

**Fig. 4.**
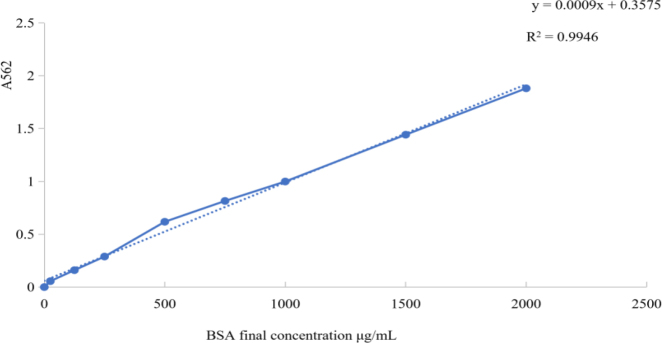
Bicinchoninic acid assay standard curve results after experimental infection of goslings with a *Mycoplasma gallisepticum* strain previously isolated from geese. A562 – absorbance at 562nm; R^2^ – coefficient of determination; BSA – bovine serum albumin

### SDS-PAGE

The SDS-PAGE results of membrane proteins showed that the goose-derived MG-GD01/22 yielded 25 protein bands, with three distinct bands at 110 kDa, 70 kDa, and 50 kDa. The chicken-derived MG R(low) yielded 23 protein bands, with the major differences concentrated in proteins with molecular weights between 25 kDa and 50 kDa (Fig. 5).

**Fig. 5.**
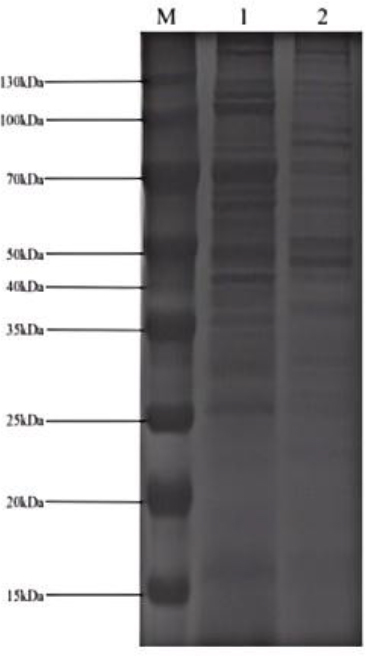
SDS-PAGE image after experimental infection of goslings with a *Mycoplasma gallisepticum* (MG) strain previously isolated from geese (MG-GD01/22) or with a strain previously isolated from chickens (MG R(low)). M – marker; 1 – MG-GD01/22; 2 – MG R(low)

### Hyperimmune serum preparation

The antibody titres of the collected goose sera were determined by HI tests. The antibody titres of negative sera were less than 1:40, whereas the MG-GD01/22 hyperimmune sera titres for geese were 1:640.

### Western blot

Western blot tests were conducted with negative sera from geese to ensure the absence of non-specific immune reactions. The results showed no reactive bands with MG-GD01/22 or MG R(low), confirming the specificity of the immune response (Fig. 6).

**Fig. 6.**
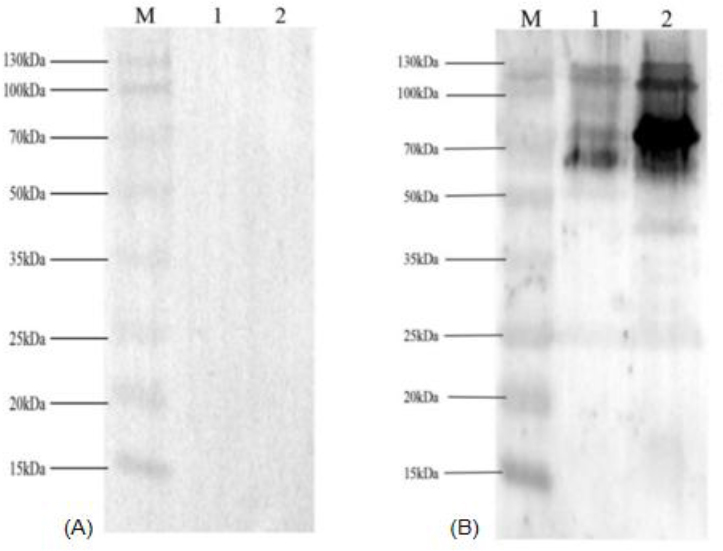
Western blot images of sera from uninfected goslings and goslings experimentally infected with *Mycoplasma gallisepticum* (MG). (A) Goose negative serum; M – marker; 1 – chicken-derived MG R(low); 2 – goose-derived MG-GD01/22. (B) Goose positive serum; M – marker; 1 – MG R(low); 2 – MG-GD01/22

Western blot assays were performed using hyperimmune sera from geese against the membrane proteins of the MG-GD01/22 and MG R(low) strains. The sera from geese recognised nine protein bands in the MG-GD01/22 membrane proteins, specifically at molecular weights of approximately 130, 110, 75, 67, 40, 35, 29, 26 and 25 kDa. Conversely, in the MG R(low) membrane proteins, the sera showed six protein bands which were located at approximately 130, 90, 75, 67, 50 and 25 kDa. Notably, the 75 kDa protein of MG-GD01/22 demonstrated pronounced immunogenicity, showing the most intense and widest band. Proteins at 130, 110 and 67 kDa also exhibited distinct immunogenic bands, while proteins at 35, 29, 26, and 25 kDa were faint, with nearly invisible bands. For the MG R(low) strain, proteins at 130, 75 and 67 kDa provoked strong immune responses, with the 67 kDa band appearing most pronounced. Proteins at 90, 50 and 25 kDa were minimal, with nearly undetectable bands.

These results demonstrate that MG-GD01/22 induces robust immune responses in geese, with the 75 kDa membrane protein exhibiting high immunogenicity. Both MG-GD01/22 and MG R(low) strains elicited significant immune reactions at 75 kDa and 67 kDa in geese, as illustrated in Fig. 6.

## Discussion

In this study, goose-derived MG-GD01/22 and chicken-derived MG R(low) were used to infect goslings *via* nasal drops and tracheal inoculation. Based on clinical symptoms and qPCR detection results, both strains showed an infection rate of over 80%, confirming the successful establishment of the MG infection model. Each of the strains was pathogenic to geese. Mahmmoud *et al*. (20) reported that MG PCR positivity in broiler flocks could reach approximately 85%, indicating widespread colonisation and successful establishment of infection in the respiratory tract. These findings suggest that high infection rates are a common feature of MG infection in chickens. Comparative analysis between Group A goslings, which were infected with MG-GD01/22 and Group B goslings, which were infected with MG R(low) revealed differences. Notably, two goslings in the MG-GD01/22 group succumbed to the infection, making a mortality rate of 20%, and contrasting with the 0% rate in the MG R(low) group. The clinical symptoms and air-sac lesion gradings in the remaining goslings and those of the MG R(low) group were similar. According to Gharaibeh and Hailat (9), experimental inoculation of chickens with MG resulted in universal infection, as demonstrated by bacteriological isolation and PCR detection. However, the clinical course was generally mild, and mortality was negligible. These observations reinforce the view that, although MG readily establishes infection in chickens, it does not commonly lead to substantial mortality in single-pathogen experimental models. Overall, these comparisons suggest that although MG demonstrates similarly high infectivity in both chickens and geese, disease severity and mortality may differ between host species. The occurrence of 20% mortality in goslings highlights a potentially greater susceptibility of geese or increased virulence of the MG-GD01/22 strain under the present experimental conditions.

Histopathological examination of tracheal and lung tissues of all groups consistently revealed cilia loss in the tracheal epithelium and minor haemorrhage in the mucosal lamina propria. Lung tissues showed typical signs of *Mycoplasma* pneumonia. Vascular haemorrhage in the pulmonary interstitium was distinctly observed in goslings infected with MG-GD01/22. These findings are consistent with previous reports describing MG-induced epithelial damage and inflammatory responses in avian species. Dykstra *et al*. (6) demonstrated desquamation of ciliated epithelial cells and epithelial thickening associated with inflammatory infiltration and oedema. Similarly, Kulappu Arachchige *et al*. (16) observed severe air-sac lesions and marked tracheal lymphocytic infiltration in chickens infected with virulent MG strains, and Zhong (32) reported rapid ciliostasis during early infection. The observed ciliary loss indicates substantial impairment of mucociliary clearance. Moreover, the pronounced pulmonary haemorrhage in goslings infected with MG-GD01/22 may suggest enhanced virulence or host-specific pathological responses in geese. Overall, our results support the conserved pathogenic mechanisms of MG, characterised by epithelial injury and inflammatory infiltration, while indicating potential interspecies variation in disease severity.

Previous whole-genome analysis of MG-GD01/22 by our group revealed high genomic homology with chicken-derived MG strains but poor collinearity, indicating substantial gene rearrangements. This genomic structural instability – particularly insertions, deletions, or inversions in regions encoding membrane proteins – is a key driver of antigenic variation and functional divergence in membrane proteins. These findings suggest that membrane proteins in goose-origin MG may exhibit significant differences (33). Analysis by SDS-PAGE of MG-GD01/22 membrane proteins identified 25 protein bands, with prominent bands at 110 kDa, 70 kDa and 50 kDa, marking the major membrane proteins. Bands ranging from 50 kDa to 130 kDa were consistent across both strains, suggesting stability and potential species-related uniformity. However, bands between 25 kDa and 50 kDa displayed significant differences, likely reflecting genetic evolution and regional variations of the pathogen. These findings are consistent with previous SDS-PAGE based characterisations of MG protein profiles. Markham *et al*. (21) identified a major surface haemagglutinin protein (pMGA) of approximately 67 kDa in strain S6 and demonstrated strain-specific molecular weight variation (67 kDa in S6 versus 75 kDa in the F strain) by SDS-PAGE and Western blot. Similarly, Ferraz *et al*. (8) reported a distinct 75 kDa protein band (p75) specific to the F-K810 vaccine strain, absent in S6(208), and further detected subtle quantitative differences in protein expression under varying culture conditions using densitometric analysis. Collectively, these observations support the coexistence of conserved immunodominant proteins and strain-specific membrane protein variability in MG. Such diversity likely arises from underlying genetic rearrangements, functional specialisation of surface proteins, and adaptive responses to environmental or host-associated pressures (8, 21).

The Western blot results demonstrate that MG-GD01/22 antisera can recognise both MG-GD01/22 and MG R(low) membrane proteins, indicating antigenic variation on the pathogen's membrane. Notably, the 75 kDa protein of MG-GD01/22, possibly a haemagglutinin protein, shows high immunogenicity, meriting further investigation (2). Studies have established that the P67 (P64) protein is a key surface antigen of MG (13), known for its strong immunogenicity and haemagglutinating properties, and that it is identical to pMG1.2 (VlhA3.03), which serves as an ELISA coating antigen (1, 3, 15, 22). This study demonstrates the pronounced immunogenicity of proteins at this molecular weight in geese. Significant species-specific reactions to the 25 kDa protein suggest potential limitations in using it as an ELISA-coating antigen for an assay for goose samples. These findings not only provide potential targets for the diagnosis of MG but also offer important insights for the development of vaccines targeting goose-derived MG in the future.

## Conclusion

This study confirms the pathogenicity of MG-GD01/22 and MG R(low) in geese, with MG-GD01/22 exhibiting enhanced virulence and lethality in geese. Comparative analysis of membrane proteins revealed that while protein bands between 50 and 130 kDa are consistent, significant differences exist in the 25–50 kDa range. Both strains elicited immune responses in geese, particularly at the 75 kDa and 67 kDa protein sizes, indicating strong immunogenicity. Given these findings, MG-GD01/22 holds potential as a candidate for further development of diagnostic kits and vaccines against MG infection in geese.
